# Early Detection of Tomato Spotted Wilt Virus by Hyperspectral Imaging and Outlier Removal Auxiliary Classifier Generative Adversarial Nets (OR-AC-GAN)

**DOI:** 10.1038/s41598-019-40066-y

**Published:** 2019-03-13

**Authors:** Dongyi Wang, Robert Vinson, Maxwell Holmes, Gary Seibel, Avital Bechar, Shimon Nof, Yang Tao

**Affiliations:** 1 Bio-Imaging and Machine Vision Lab, Fischell Department of Bioengineering, University of Maryland, College Park, MD 20742 USA; 2 The Institute of Agriculture Engineering, Agriculture Research Organization, Volcani Center, P.O.Box 6, Bet Dagen, 50250 Israel; 3 School of Industrial Engineering, Purdue University, 315N Grant Street, West Lafayette, IN 47907-2023 USA

## Abstract

Tomato spotted wilt virus is a wide-spread plant disease in the world. It can threaten thousands of plants with a persistent and propagative manner. Early disease detection is expected to be able to control the disease spread, to facilitate management practice, and further to guarantee accompanying economic benefits. Hyperspectral imaging, a powerful remote sensing tool, has been widely applied in different science fields, especially in plant science domain. Rich spectral information makes disease detection possible before visible disease symptoms showing up. In the paper, a new hyperspectral analysis proximal sensing method based on generative adversarial nets (GAN) is proposed, named as outlier removal auxiliary classifier generative adversarial nets (OR-AC-GAN). It is an all-in-one method, which integrates the tasks of plant segmentation, spectrum classification and image classification. The model focuses on image pixels, which can effectively visualize potential plant disease positions, and keep experts’ attention on these diseased pixels. Meanwhile, this new model can improve the performances of classic spectrum band selection methods, including the maximum variance principle component analysis (MVPCA), fast density-peak-based clustering, and similarity-based unsupervised band selection. Selecting spectrum wavebands reasonably is an important preprocessing step in spectroscopy/hyperspectral analysis applications, which can reduce the computation time for potential in-field applications, affect the prediction results and make the hyperspectral analysis results explainable. In the experiment, the hyperspectral reflectance imaging system covers the spectral range from 395 nm to 1005 nm. The proprosed model makes use of 83 bands to do the analysis. The plant level classification accuracy gets 96.25% before visible symptoms shows up. The pixel prediction false positive rate in healthy plants gets as low as 1.47%. Combining the OR-AC-GAN with three existing band selection algorithms, the performance of these band selection models can be significantly improved. Among them, MVPCA can leverage only 8 spectrum bands to get the same plant level classification accuracy as OR-AC-GAN, and the pixel prediction false positive rate in healthy plants is 1.57%, which is also comparable to OR-AC-GAN. This new model can be potentially transferred to other plant diseases detection applications. Its property to boost the performance of existing band selection methods can also accelerate the in-field applications of hyperspectral imaging technology.

## Introduction

Tomato spotted wilt virus (TSWV) is one of the common threats to more than 1,000 plant species from different botanical families^1^. It can cause a range of symptoms in a persistent and propagative manner, including sudden yellowing, mild mottling, mosaic and so on^2^. In practice, once the symptoms start developing, it is too late to head off an epidemic^3^. Since 2004, TSWV isolates have overcome the resistance gene Tsw in pepper^4^, making it more difficult to manage. Bell pepper is a high-value specialty crop grown mostly in greenhouses for fresh markets. It is cultivated worldwide and used as a food ingredient, spice and ingredient in medicine. Therefore, early detection of TSWV is a crucial issue to ensure all the infected pepper plants eradicated as soon as possible^5^.

Monitoring plant health and detecting pathogens effectively are important topics in precision agriculture research^6^. Early detection is meaningful to reduce disease spread and to facilitate management practice^7^. Molecular-level direct detection method can accurately evaluate the plant disease levels, but it’s hard to be conducted in real-time field test scientific^8^. Comparatively, machine vision-based indirect detection method is more attractive in practice because of their non-invasive properties and their abilities to identify plant diseases through various parameters including color^9^, morphological^10^ and temperature changes^11^. Hyperspectral imaging (HSI) makes use of the plants’ interaction with different electromagnetic spectra, and forms an image containing the intrinsic information of the leaf biochemical compounds and leaf anatomical structure^11^. Compared to RGB color imaging system, HSI including near infrared information makes early plant disease detection possible, and the subtle changes in spectral reflectance of plants could reflect early and invisible symptoms of the disease^12^. This technology has achieved great success in analyzing chemical component levels^13,14^ and plant diseases^3,12,15^.

However, problems which potentially constraint the wide applications of HSI in early stage plant disease detection remain to be solved. Firstly, most advanced HSI algorithms were developed and validated based on some public remote sensing dataset^16,17^. Furthermore, for early stage plant disease detection applications, it is very hard to get a well-prepared dataset, especially the pixel-level ground truth for invisible disease symptoms. Even experienced experts cannot label where the invisible disease symptoms are and define the pure invisible diseased pixel, which is important for some HSI analysis methods^18,19^. Most current research takes the plant as a whole, and the average plant spectrum is used for plant classification^20^, whereas in practice, mean spectrum can hardly represent the whole plant. Different illumination conditions can make the spectrum of different plant locations vary significantly. For diseased plants in the early stage, the diseased spot may be small, and computing the average spectrum could wipe out the diseased symptoms. Meanwhile, because there is no prior knowledge of the spatial distribution of invisible symptoms, it is difficult to make use of spatial information to improve the HSI analysis performances though the strategy is very effective in many other HSI applications^19,21,22^. Therefore, determining exact spectrum characteristics is crucially important for hyperspectral image analysis and its applications for early plant disease detection.

Secondly, classifying healthy and diseased spectrums is not a trivial task because of the spectrum similarity and imaging system noise. Full spectra information may benefit the discrimination performance because there is no information loss, but meanwhile, it rapidly increases the complexity of modelling caused by information redundancy, especially with the required computation power and the analysis time in practice^23^. Decreasing the features number of the spectral signal is the common solution to the problem using techniques like projections^24^, clustering^25–27^, or autoencoders^28^. The extracted features can be sent into a discrimination model like linear discriminant analysis or support vector machine, to do classification^29,30^. However, some of the dimensional reduction processes sacrifice the original physical meaning of spectral signals due to some linear or non-linear transformations^31,32^. To preserve the physical information and make the model interpretable, selecting ‘adequate’ wavebands from the original hyperspectral space^3,33,34^ is more attractive. For biological engineering applications, preserving the original band information is also very important because once the ‘adequate’ wavebands are determined, the expensive hyperspectral camera can be downgraded to more reliable and cost-effective multispectral camera which is more likely to be used in the field. There are many research on band selection algorithms, which can be divided into supervised and unsupervised methods^35–39^. Supervised band selection methods usually rely on some specific criterion functions and discrimination models^38,39^. However, for the early stage plant disease detection application, it is very difficult to quantify pixel-level classification performance of invisible diseased pixels, and thus unsupervised band selection is more promising in this specific domain. Most current unsupervised band selection models are easily affected by system noise, data crossovers and outliers^30^, and how to improve their performance is still an open topic.

Consequently, a new hyperspectral analysis model, named as outlier removal auxiliary classifier generative adversarial nets (OR-AC-GAN) is proposed in this paper. It is a variant of Generative Adversarial Network (GAN)^40^, a popular neural network architecture in deep learning domain. In recent years, the concept of deep learning has achieved great success in many areas^41,42^. In this paper, the proposed OR-AC-GAN is expected to meet the following specific objectives: (i) Without band selection, it can classify the pixels in hyperspectral images into backgrounds, diseased plant pixels and healthy plant pixels in single step, and thus the exact plant disease positions can be determined. (ii) From the plant level, it can detect diseased plants before specific symptoms show up. (iii) Compared to other models, it can effectively reduce the pixel prediction false positive rate in healthy plants without affecting the plant-level prediction result. (iv) It can generate some fake spectrum data following original data distribution. The generated data can be further used for some classic unsupervised band selection models and is expected to improve their performances. Overall, the novel method can detect the TSWV disease sensitively at an early stage before the symptom is visible making use of full hyperspectral information. It can also be potentially used in field for real-time applications because the generated data from the model can boost the performance of existing band selection algorithms and preserve the classification accuracy with limited bands.

## Results

### Outlier removal auxiliary classifier generative adversarial nets (OR-AC-GAN)

**Figure 1:**
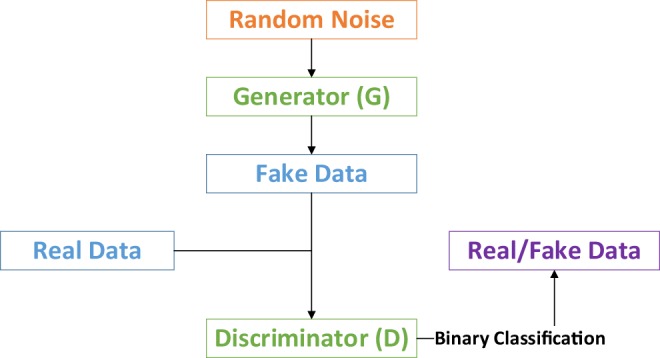
Basic architecture of GAN model.

The idea of proposed OR-AC-GAN originates from a new and promising type of generative model named generative adversarial nets (GAN), as shown in Fig. 1^40^. It can learn the data distribution from scratch, and doesn’t need any pre-knowledge and preliminary assumptions about dataset. Generally, there is a generator and a discriminator in the GAN model. The generator aims to create fake data as real as possible, and the discriminator targets for distinguishing the fake date g from the real data x. Once the GAN model is well-trained, the generator is expected to describe real data distribution, which can be used for augmenting the dataset.

**Figure 2:**
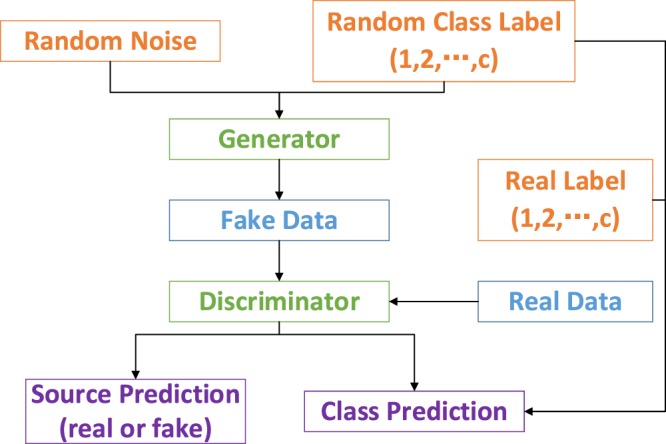
The architecture of AC-GAN model.

Auxiliary classifier GANs (AC-GAN) is a variant of GAN network^43^, which combines a c classification task into the GAN model, as shown in Fig. 2. It can effectively augment the classification dataset during the network training procedure. In addition, research shows the additional task can stablize the GAN training process^44,45^.

In practice, the Achilles’ heel of AC-GAN model is if the real distribution of two classes is very closed, the data augmentation by generator can completely ruin the classification ability of model. The reason for that is, in AC-GAN model, even if the binary discriminator determines the data as fake, the classifier still needs to allocate the data into a particular class. It is originally designed for increasing the dataset, but it also strengthens the side-effect of data outliers and crossovers. This problem becomes overwhelming in our application due to the trival spectrum differences among healthy and diseased pixels. The augmentated data of the two different classes could confuse the *D*, and further affect the generation ability of *G*.

**Figure 3:**
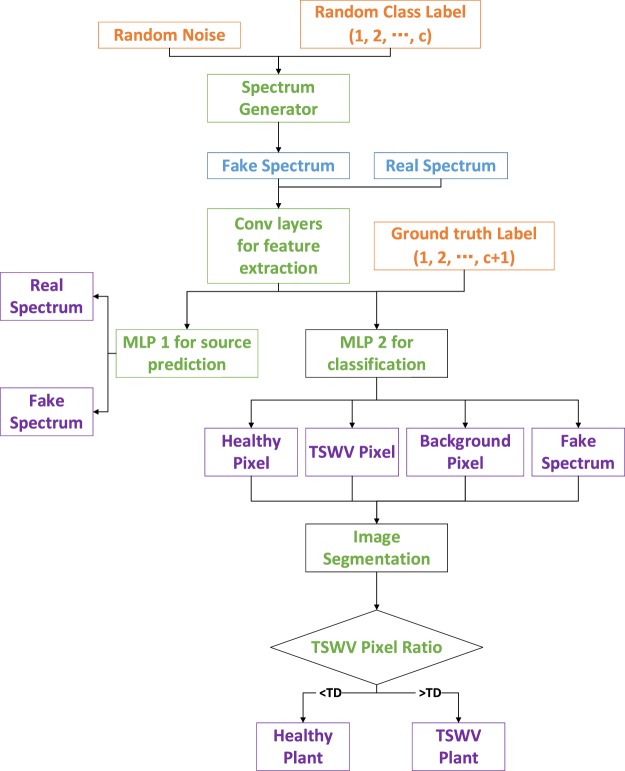
The system architecture of proposed OR-AC-GAN model and its application for early TSWV detection. MLP 1 and MLP 2 are two multi-layer perceptrons. TSWV (diseased) pixel ratio is defined as the ratio of the number of predicted TSWV pixels to the sum of predicted TSWV and healthy pixels. *TD* means a threshold to determine the plant is diseased or not based on the TSWV pixel ratio.

The proposed OR-AC-GAN made a subtle change of AC-GAN, but the inherent idea of the two models are completely different. As shown in Fig. 3, the art of OR-AC-GAN is that an additional label *c* + *1* is allocated when training the *D*, and all fake data is classified into the additional class. It means even if the fake data is closed enough to the real data, it will still be classified into the additional type. This design can obviously improve the classification criterion, rule out the data outliers, and the generated data from OR-AC-GAN can also focus on the intrinsic features of data in different classes. In the test phase, the *D* can classify the image spectrum pixels as background, healthy or TSWV, which can be used for locating the diseased positions.

**Figure 4:**
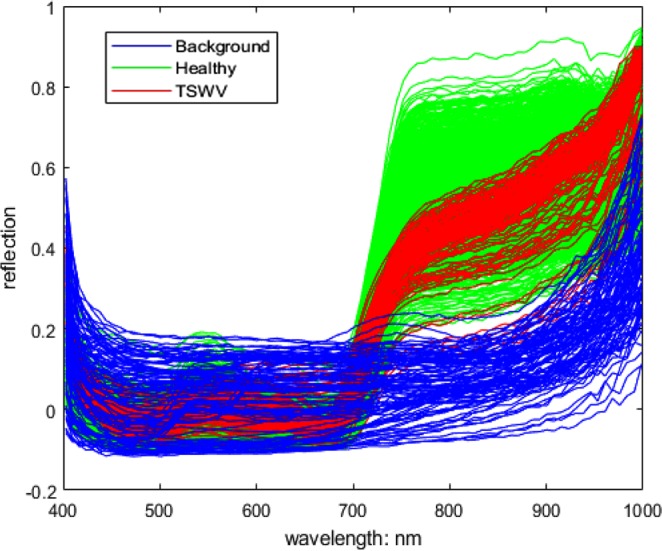
The typical real spectrums of different classes. Blue: background pixel. Green: healthy pixels. Red: TSWV pixels.

**Figure 5:**
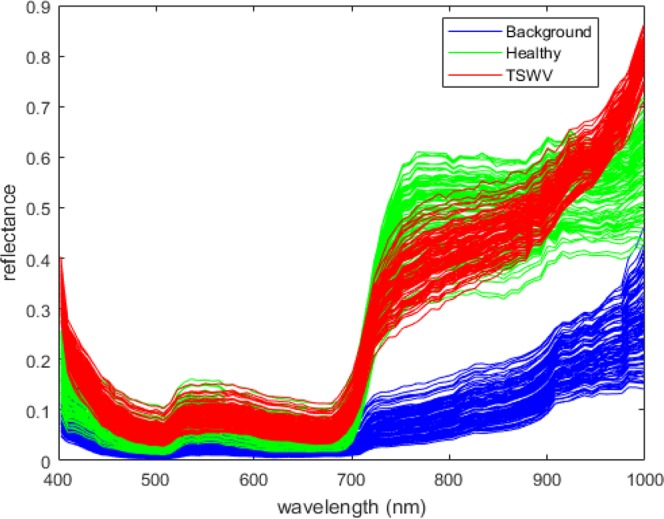
The typical generated spectrums of different classes. Blue: fake background pixels. Green: fake healthy pixels. Red: fake TSWV pixels.

**Figure 6:**
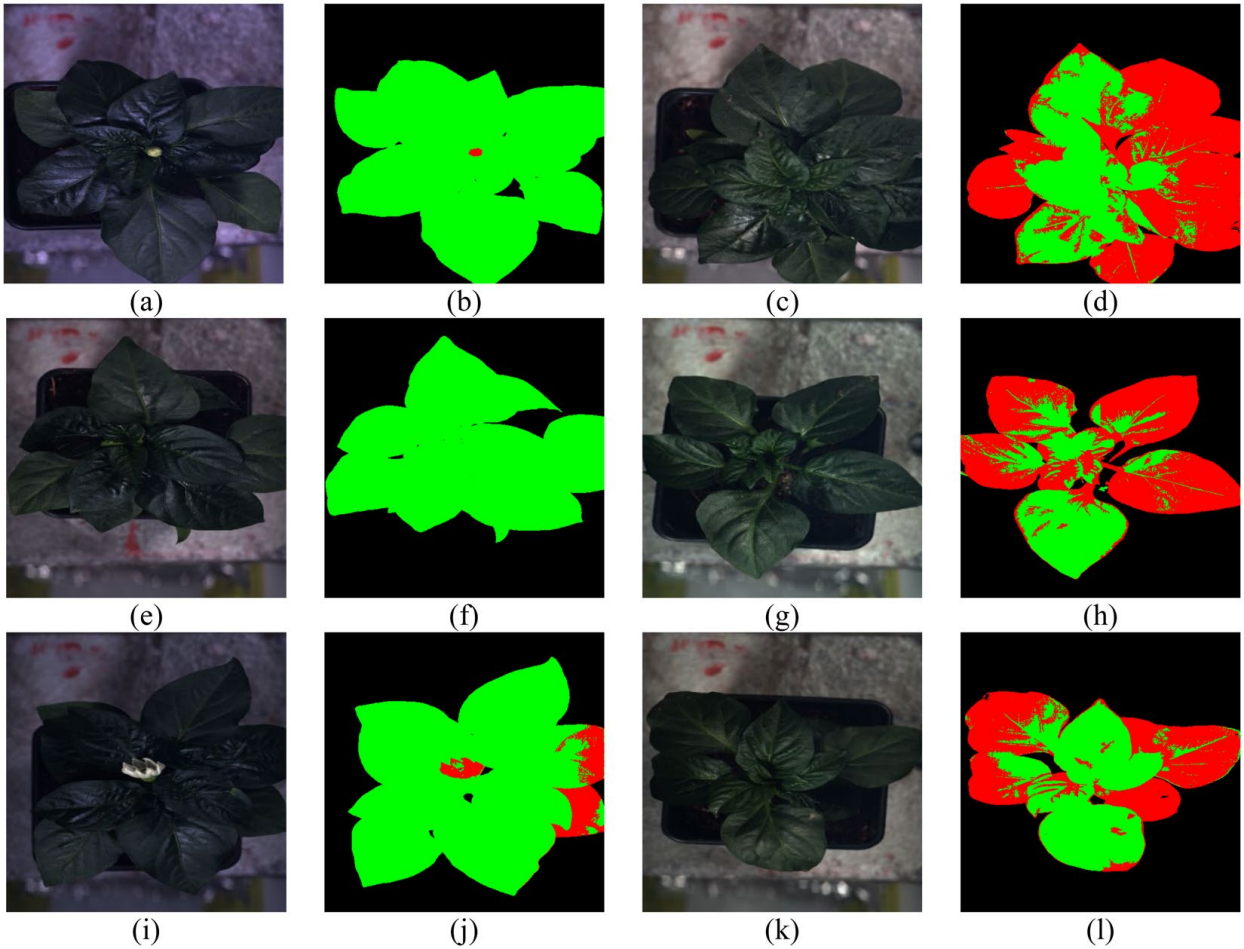
Typical results for test plants from OR-AC-GAN. Healthy pixels are labelled as green, and TSWV pixels are labelled as red. Left: (**a**,**b**), (**e**,**f**), and (**i**,**j**) are three healthy plants and their corresponding classification results. Right: (**c**,**d**), (**g**,**h**), and (**k**,**l**) are TSWV infected plants and their corresponding classification results.

Figure 4 shows some typical spectrums of healthy, TSWV and background pixels in real dataset. After the OR-AC-GAN model is well-trained, the generated spectrums which are shown in Fig. 5 can capture the intrinsic features of real spectrums in different classes. The generated spectrums improve the classification results of OR-AC-GAN and the performance of classic band selections models. The related experiment results will be shown in following sections. The well-trained discriminator can segment the hyperspectral images as described in Fig. 3. The typcial visualized classificaiton results are shown in Fig. 6, where green indicates healthy and red indicates the possible TSWV infection.

### Evaluation of pixel and plant level classification results

As shown in Fig. 6(j), there are some predicted diseased pixels in a healthy plants. To futher quantify the classification performance of model, two pixel-level and two plant-level metrics are defined here.

The first pixel-level metric is to evaluate the plant segmentation performance. The TSWV and healthy pixels are called by a joint name plant pixels. The ground truth plant pixels are labelled manually. The expression of the metric, *Acc*_pixel_, is defined in Equation 1.

1 $${{\rm{Acc}}}_{pixel}=\frac{PN{C}_{ppixel}+PN{C}_{bpixel}}{P{N}_{total}}$$

where *PNC*_*ppixel*_ is the number of plant pixels which are predicted correctly (TSWV and healthy pixels may mix up). *PNC*_bpixel_ is the number of background pixels which are predicted correctly. *PN*_*total*_ is the number of pixels in a hyperspectral image. The average *Acc*_*pixel*_ value is 98.03% for 54 plants in test dataset.

The second pixel-level index is to evaluate how well the model can distinguish the healthy pixels and TSWV pixels. It is defined as the false positive rate of TSWV pixels in the healthy plants (*FP*_*Tpixel,Healthy*_), shown in Equation 2.

2 $${F}{{P}}_{{Tpixel}{,}{Healthy}}=\frac{{P}{{N}}_{{Tpixel}{,}{Healthy}}}{{P}{{N}}_{{Tpixel}{,}{Healthy}}+{P}{{N}}_{{hpixel}{,}{Healthy}}}$$

where *PN*_*Tpixel,Healthy*_ is the number of predicted TSWV pixels in healthy plants, *PN*_*hpixel,Healthy*_ is the number of predicted healthy pixels in healthy plants. This metric is only defined in healthy plants because pixel-level ground truth is not available in the TSWV plant, but all the plant pixels in the healthy plants should be classified as healthy. For the 27 healthy plants in test dataset, the average *FP*_*Tpixel,Healthy*_ value is 1.47%, with standard derivation of 2.53%. The worst (largest) value in the test dataset is 8.21%.

The plant-level metric includes the specificity and sensitivity value based on the plant-level classification results. The definitions are shown in Equation 3.

3 $$\{\begin{array}{c}{Sensitivit}{{y}}_{{plant}}=\frac{{T}{{P}}_{{plant}}}{{T}{{P}}_{{plant}}+{F}{{N}}_{{plant}}}\\ {Specificit}{{y}}_{{plant}}=\frac{{T}{{N}}_{{plant}}}{{T}{{N}}_{{plant}}+{F}{{P}}_{{plant}}}\end{array}$$

where *TP*_*plant*_ is the true positive value. *TN*_*plant*_ is the true negative value. *FP*_*plant*_ is the false positive value, and *FN*_*plant*_ is the false negative value. All these metrics are defined in plant-level.

**Figure 7:**
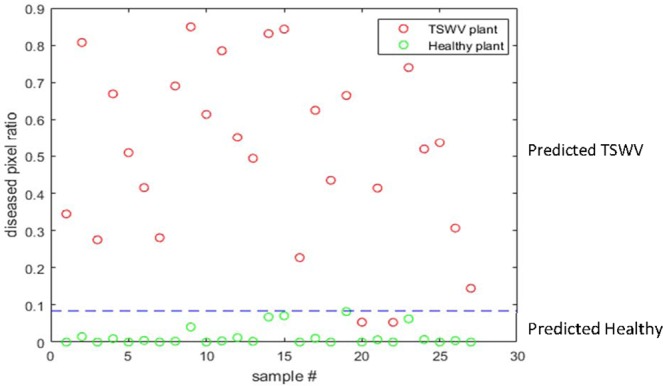
The classificaiton result of an independent test dataset, where the predicted healthy and diseased plants can be separated by the threshold *TD* determined by the algorithm in Fig. 3 based on the diseased pixel ratio of a plant.

Figure 7 shows the result of an independent test dataset of 54 plants by the algorithm in Fig. 3. In Fig. 7, the red points represent diseased plants, and the green points represent healthy plants. The blue line represents the disease threshold *TD* defined in Fig. 3. The plants with diseased pixel ration larger than *TD* are defined as TSWV plants, otherwise they are predicted as healthy plants. The *TD* value determination takes both sensitivity and specifity into consideration and aims to maximize the quadratic sum of sensitivity and specificity. In the experiment, the optimized *TD* value is 0.084 and the corresponding sensitivity and specificity value are 92.59% and 100%, respectively.

### Comparison with one dimensional convolutional neural network (1D-CNN) and AC-GAN

One dimensional CNN has been a powerful and promising method in spectrum analysis domain in recent years^46,47^. However, in the plant diseases detection applications, imbalance number diseased and healthy pixels can affect the classification results. As mentioned earlier, AC-GAN can augment the classification dataset, but it can also amplify the outliers in the dataset. The proposed OR-AC-GAN aims to solve the problems in the two current models. With the same network configurations, the comparison results of three models are shown in Table 1.

**Table 1:** Statistic comparison results of different network architectures.

	*Acc*_*pixel*_	*FP*_*Tpixel,Healthy*_ (average)	*FP*_*Tpixel,Healthy*_ (standard derivation)	*FP*_*Tpixel,Healthy*_ (worst)	*Sensitivity*_*plant*_	*Specificity*_*plant*_
1D-CNN	98.02%	5.95%	14.79%	34.75%	**92.59%**	92.59%
AC-GAN	98.00%	10.41%	15.32%	57.59%	88.89%	**100%**
OR-AC-GAN	**98.03%**	**1.47%**	**2.53%**	**8.21%**	**92.59%**	**100%**

**Figure 8:**
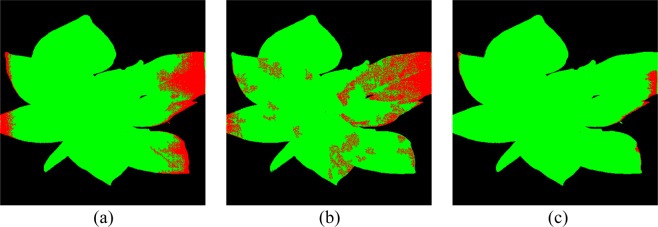
Comparison of segmentation results of a typical healthy plant. (**a**) direct CNN model. (**b**) AC-GAN model. (**c**) the proposed OR-AC-GAN model.

For *Acc*_*pixel*_, there are no significant differences among three models, and all of them get >98% classification accuracy. It means the three models can successfully distinguish plant pixels and background pixels. For *FP*_*Tpixel,healthy*_, healthier pixels are regarded as diseased pixels in AC-GAN than 1D-CNN proving the statement that AC-GAN can intensify the side-effects of outliers, and decrease the discrimination ability of model. On the contrary, the OR-AC-GAN can weaken the side-effects and augment the dataset online. Figure 8 shows the segmentation result of a typical healthy plant based on the three models, which supports the data shown in Table 1.

### Improve the performances of classic band selection algorithms

Three classic unsupervised band selection algorithms are tested here, including maximum variance principle component analysis (MVPCA)^37^, fast density peak-based clustering (FDPC)^36^ and similarity-based unsupervised band selection (SUBS)^35^ models. The experiment wants to prove OR-AC-GAN can improve the performance of the band selection models. All these models are firstly applied based on the real spectrum dataset. Then the dataset is doubled by adding the fake spectrums from OR-AC-GAN, and all the band selection algorithms are conducted again based on the augmented dataset. Once the spectrum bands are determined, the pixels will be classified as background, TSWV or healthy pixels via the k-nearest neighbor algorithm (KNN)^48^. KNN is a classic machine learning algorithm which has been widely applied in scientific and engineering domain^49,50^.

**Figure 9:**
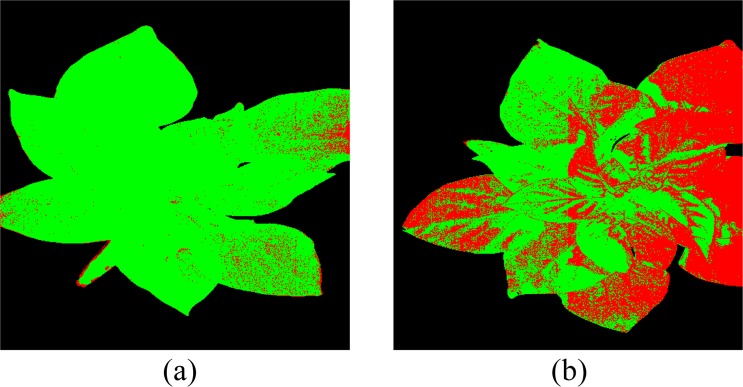
Typical visible prediction results from OR-AC-GAN + MVPCA + KNN. (**a**) A healthy plant (**b**) a TSWV plant.

Table 2 shows how the number of selected bands affect the average value of *FP*_*Tpixel,healthy*_ in different band selection models. Generally, the average *FP*_*Tpixel,healthy*_ value decreases with more bands are selected for classification. Compared to applying the band selection on the original dataset, the band selection based on both original and fake dataset can effectively lower the *FP*_*Tpixel,healthy*_ value. This trend usually shows up with more spectrum bands are selected, because the data distributions from OR-AC-GAN are more concentrated and KNN can’t get effective information from one or two bands of the refined data. Among the three classic band selection models, MCPCA gets smallest *FP*_*Tpixel,healthy*_ value (1.57%), which is comparable to the OR-AC-GAN result (1.47%). Eight bands are selected by MVPCA, which are 522 nm, 572 nm, 629 nm, 643 nm, 694 nm, 797 nm, 804 nm and 908 nm. The KNN model considers 4 nearest neighbors in this case. The plant segmentation result of typical plants are shown in Fig. 9. The *Acc*_*pixel*_ value is 97.38%, also comparable to the 98.03% from OR-AC-GAN. For the plant-level, the *Sensitivity*_*plant*_ and *Specificity*_*plant*_ values are same as the OR-AC-GAN results, which are 92.59% and 100%, respectively.

**Table 2:** The pixel-level false positive rate (*FP*_*Tpixel,Healthy*_) of MVPCA, FDPC, SUBS, OR-AC-GAN + MVPCA, OR-AC-GAN + FDPC and OR-AC-GAN + SUBS.

Band Number	1	2	3	4	5	6	7	8
MVPCA	0.430	0.336	0.214	0.143	0.139	0.115	0.112	0.123
OR-AC-GAN + MVPCA	0.542	0.151	0.146	0.101	0.053	0.066	0.017	**0.016**
FDPC	0.175	0.158	0.148	0.106	0.089	0.075	0.083	0.088
OR-AC-GAN + FDPC	0.091	0.091	0.090	0.076	0.077	0.071	0.070	**0.069**
SUBS	N/A	0.204	0.081	0.084	0.077	0.078	0.078	0.080
OR-AC-GAN + SUBS	N/A	0.245	0.073	0.074	0.074	0.070	**0.063**	0.071

### From the view of timelines

**Figure 10:**
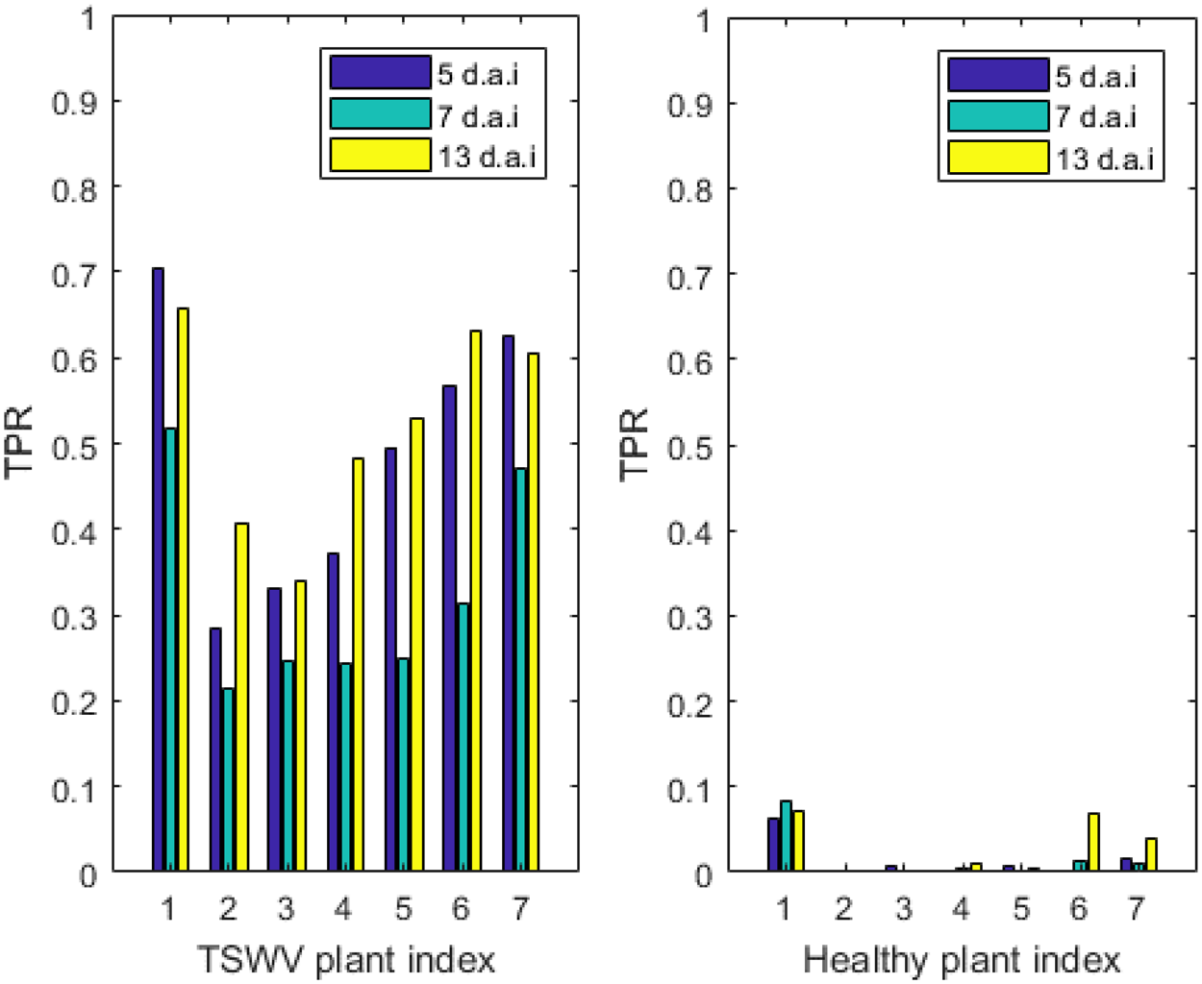
The TSWV diseased pixel ratio to the plant pixels (TPR value) for TSWV and healthy plants in different d.a.i. The three TSWV plants and three healthy plants in the training dataset are not included in the figure.

The hyperspectral images are taken 5 day after inoculate (d.a.i), 7 d.a.i and 13 d.a.i separately. In this section, the analysis for OR-AC-GAN will be conducted from the view of timeline. The changes of the TSWV diseased pixel ratio to the plants (excluding the plants used for training) pixel (*TPR*) with time are shown in Fig. 10. The *TPR* for healthy plants is same as the definition of *FP*_*Tpixel,healthy*_.

In the figure, the differences among healthy and TSWV plants are obvious and have been discussed in previous sections. The *TPR* values of both healthy and TSWV plants maintain the consistency in different d.a.i. For TSWV plants, the images from 13 d.a.i show relatively high *TPR* compared to the images from 5 and 7 d.a.i. The *TPR* relationships of 5 and 7 d.a.i images are upside-down compared to the expectation. There are couple of potential reasons to explain the problem. Firstly, there are some new leaves showing up in 7 d.a.i, which could block the old leaves and directly affect the *TPR* value. Secondly, other research^20^ shows, for the particular plant disease, there are some crossovers of hyperspectral spectrums in different d.a.i, and TSWV could have similar phenotypes in spectrum domain. Thirdly, in network training process, the time information is not included in the pixel-level ground truth, because during the data labelling process, human experience can only determine the plant status according to visible disease symptoms. Lastly, illumination condition could affect the classification result. Further research will continue to explain the phenomenon deeply. Nevertheless, the OR-AC-GAN model has revealed the significant comparative difference between the diseased and healthy as early as 5 d.a.i.

## Discussion

Targeting for the early stage plant disease detection applications, a new hyperspectral analysis model, OR-AC-GAN, is proposed. In this report, a wide-spread plant disease TSWV is used for validating the model. The pixel-level classification false positive rate in healthy plants can achieve as low as 1.47%. The plant-level classification sensitivity and specificity can get 92.59% and 100%. The average classification accuracy is 96.25%.

Compared to existing research^12,20,51,52^, the proposed model have serveral advantages. Firstly, traditional analysis models need firstly determine region of interests in images and extract ‘reasonable’ image features, including spectrum bands and spatial features. All these information was sent into a classifier to conduct pixel-level or plant-level classification. For different environment and experiment objects, the analytical strategies could vary a lot. On the contrary, OR-AC-GAN is a relatively fixed all-in-one model, which successfully integrates the task of image segmentation, feature extraction and classification.

Secondly, compared to applying CNN model directly, OR-AC-GAN successfully augment the dataset online, especially the data of diseased pixels. Compared to the AC-GAN model, it can automatically remove the outliers in the dataset, and avoid the decreasing discrimination ability accompanying with the data augmentation.

Thirdly, the well-trained OR-AC-GAN model is able to generate fake spectrums from random noises. Combining with the real spectrums, the fake spectrums improve the robustness and performance of some classic band selection methods. The trend becomes remarkable with the increasing of the number of selected spectrum bands. In the experiment, three band selection methods are tested, including MVPCA, FDPC and SUBS. Among them, MVPCA shows the best performance. It uses eight wavelengths to get comparable results as OR-AC-GAN which utilizes 83 wavebands. This experiment is meaningful from both the scientific and engineering view. Pre-determination of the spectrum bands can not only cut the cost of analytical system but also make the hyperspectral model explainable from the scientific view. For example, the eight bands selected by MVPCA are highly related to the photosynthetic capacity and red inflection point according to other research^53^.

Our further research will focus on the view of timeline, and observe how hyperspectral images changes with time before visible symptoms in plants show up. Currently, the experiment only proves TSWV is distinguishable as early as 5 d.a.i. Meanwhile, different diseases will be tested to prove the robustness of the model. To further improve the early stage disease detection efficiency, other information like leaf temperature, chlorophyll content is expected to be integrated into the proposed model.

## Methods

### Image dataset construction

**Figure 11:**
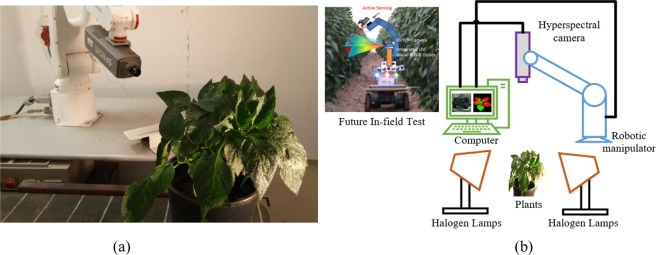
(**a**) The real experiment imaging station. (**b**) The schematic diagram of the image stataion. The system is expected to be mounted in the agriculture robotic for in-filed plant disease detection.

Plants of sweet pepper (Hazera Genetics) were obtained from a commercial nursery (Hishtil, Ashkelon, Israel) 40–50 days after seeding and were transplanted into 20 pots containing soil and potting medium and were fertigated proportionally with drippers 2–3 times per day with 5:3:8 NPK fertilizer (nitrogen (N), phosphorus (P) and potassium (K)), allowing for 25–50% drainage. Ten healthy plants (control), 10 plants infected with TSWV. Infecting the diseases were conducted and controlled by a plant pathologist. Images of the top part of all plants were acquired at a laboratory with hyperspectral camera (400–1000 nm, V10E Specim ImSpector) mounted on a Motorman 5L robotic manipulator as shown in Fig. 11(a). Two halogen lamps were placed within 0.5 m from the examined plant with a vertical orientation of 45 degrees as light sources. The schematic of the imaging system is shown in Fig. 11(b). Hyperspectral images were taken 5 d.a.i. (days after infection), 7 d.a.i. and 13 d.a.i, and total 60 images are in the dataset. All the images are calibrated based on the white and dark reference image^20^.

### Network training procedure

The dataset consists 60 hyperspectral images, 30 of which are healthy plants images, and the left 30 are TSWV plants images plants. To train the OR-AC-GAN model, a pixel-level spectrum training dataset needs to be prepared. Because most of TSWV plants haven’t shown visible symptoms in RGB images, the three TSWV plant images in the training dataset need to be well-chosen and the diseased spots in the selected plants should be visible. In the experiment, three training TSWV images are selected from the data of 13 d.a.i. The diseased pixels in the plants are manually labelled and added into the pixel-level training dataset. On the contrary, there is no specific requirement for selecting training healthy plants. In the experiment, three healthy plants are randomly selected from the data of 5 d.a.i., 7 d.a.i., 13 d.a.i, respectively. To rule out the spectrum differences resulting from different illumination conditions, the pixels in both the common illumination and the shadow are expected to be included in the training dataset. Background pixels in the pixel-level training dataset are selected from three TSWV images and three healthy images mentioned above. In total, there are 103,769 background pixels, 105,561 healthy pixels and 2,071 TSWV pixels in the pixel-level training dataset. The typical spectrums of the three type of pixels in shown Fig. 4. The remaining 54 images are used for algorithm test.

**Figure 12:**
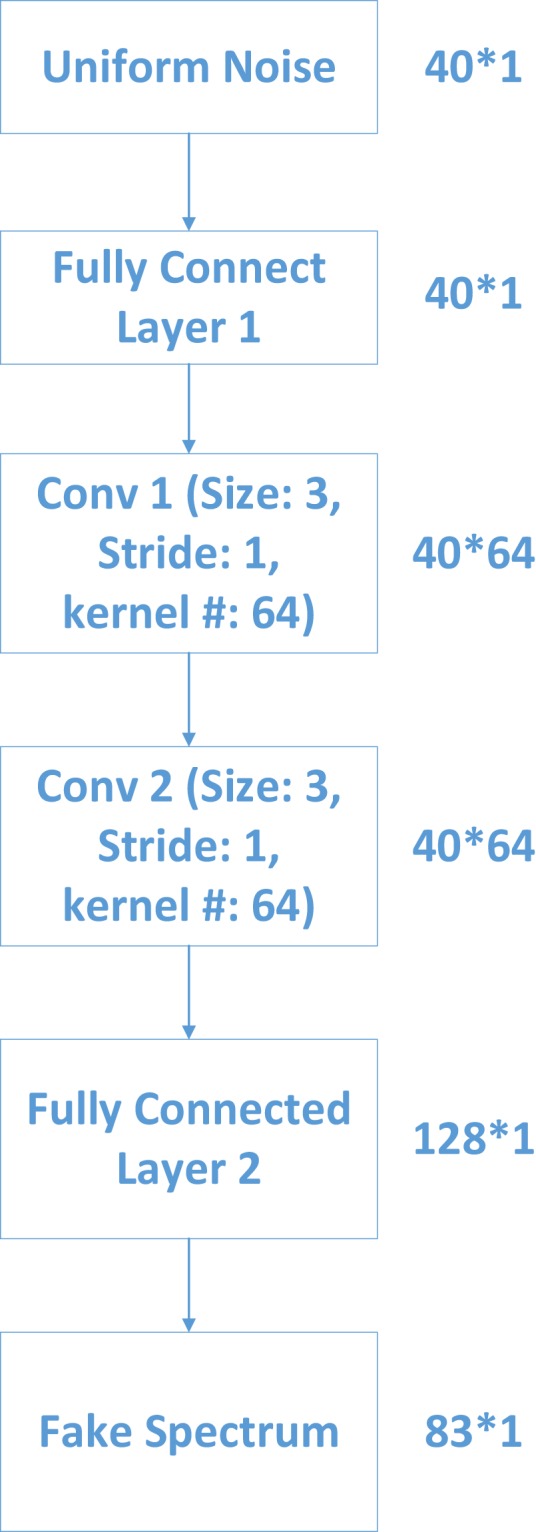
The detailed generator structure in proposed OR-AC-GAN model.

The well-prepared pixel-level dataset is used for training OR-AC-GAN model. The inputs of generator in OR-AC-GAN model are 40 random numbers ranging from 0 to 1 which follow the uniform distribution. The random noise passes through a series of fully connected layers, convolutional layers and non-linear operations to acquire a fake spectrum vector with 83 bands. The detailed structure of generator is shown in Fig. 12.

**Figure 13:**
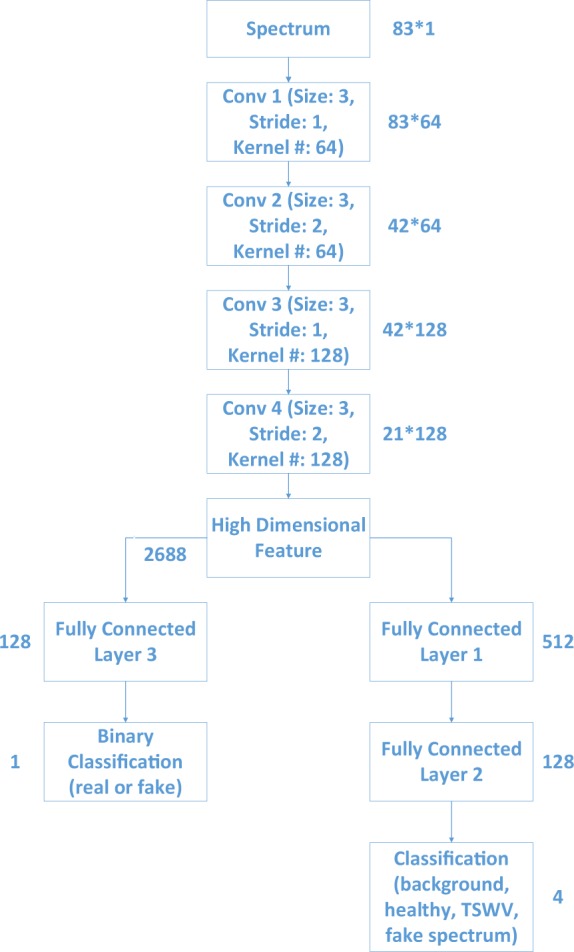
The detailed discriminator structure in proposed OR-AC-GAN model.

Both the generated spectrum and real spectrum are fed into the discrimator in OR-AC-GAN model. The discrimator is composed of several one-dimensional convolutional layers to extract data features. These features are sent into multiple layer perceptron (MLP) in parallel for two different tasks, source prediction and classification. The MLP for source prediction is equipped with only one fully connected layer and the MLP for classification has two fully connected layers in series. This design is to separate the two tasks based on the degree of difficulties. The detailed structure of discrimator is shown in Fig. 13.

Define the log-likelihood of source prediction as *L*_*S*_, the log likelihood of classification in *D* training process as *L*_*C*1_, and the log-likelihood of classification in G training process as *L*_*C2*_. The definitions of *L*_*S*_, *L*_*C1*_ and *L*_*C2*_ are shown in Equation 4. When *D* is trained, the network’s target is to maximize *L*_*S*_ + *L*_*C1*_. It aims to determine the fake data as fake and real data as real. Meanwhile, it needs to classify the data into correct classes. Real data is classified according to the labelled ground truth, and the fake data is classified into the additional class *c* + *1*. When training *G*, the network’s target is to maximize *L*_*C2*_ − *L*_*S*_. The role of *L*_*C2*_ is to classify the real data corresponding to the labelled ground truth, same as *L*_*C1*_. However, it also needs to classifty the fake data according to the random class labels fed into the generator.

4 $$\{\begin{array}{c}{{L}}_{{S}}={{E}}_{{x} \sim {{p}}_{{x}}}[\,\mathrm{log}\,{P}({S}={real}|x)]+{{E}}_{{z} \sim {{p}}_{{z}}}[\,\mathrm{log}\,{P}({S}={fake}|G({z}))]\\ {{L}}_{{C1}}={{E}}_{{x} \sim {{p}}_{{x}}}[\,\mathrm{log}\,{P}({C}={{c}}_{{real}}|x)]+{{E}}_{{z} \sim {{p}}_{{z}}}[\,\mathrm{log}\,{P}({C}={c}+1|{G}({z}))]\\ {{L}}_{{C2}}={{E}}_{{x} \sim {{p}}_{{x}}}[\,\mathrm{log}\,{P}({C}={{c}}_{{real}}|x)]+{{E}}_{{z} \sim {{p}}_{{z}}}[\,\mathrm{log}\,{P}({C}={{c}}_{{fake}}|G({z}))]\end{array}$$

The training process is based on the principle of back propagation and gradient descent algorithm^48^. The learning rate is updated based on Adam method^54^. Due to the imbalance of the dataset, the class weights are adjusted based on the number of training pixels in different class. After 50-epoch training, the output of generator is visualized as in Fig. 5. The deep learning codes are implemented by the Python keras library^55^ with Nvidia GeForce GTX Titan Xp GPU.

### Band Selection

OR-AC-GAN model utilizes 83 spectrum bands information to do the pixel-level classification. However, in practice, if some spectrum bands can be selected in advance, the analysis model will be dramatically simplified, and the hyperspectral spectral imaging system can also be degraded into multispectral imaging system to cut the cost.

There are classic hyperspectral bands selection algorithms posed in remote sensing field. The main target of band selection criterial is to reduce the data redundancy and to select representative examplars. However, these band selection methods are highly relied on the data quality. Similar spectrums and data outliers can affect the performances of band selection algorithms. As mentioned earlier, a well-trained OC-AC-GAN model can remove data outliers online, and it is expected to improve the performance of three classic band selection models, including MVPCA^37^, FDPC^36^ and SUBS^35^. The descriptions of the three models are listed below. Assuming there are *N* one-dimensional spectrum data with *l* bands. The *s*_*ij*_ means the jth band of *ith* data sample.

### Maximum variance principle component analysis (MVPCA)

MVPCA^37^ is a joint band prioritization and band decorrelation approach. It ranks the bands by a criterion that comprises the importance of an individual band and its correlation with other bands^36^.

Assume ${\rm{\Sigma }}=1/{N}{\sum }_{{i}={1}}^{{N}}({{s}}_{{i}}-{m}){({{s}}_{{i}}-{m})}^{{T}}$ is the covariance matrix of spectrum data, where m is the sample mean vector. The importances of each band is defined in Equation 5.

5 $$Weigh{t}_{k}={\sum }_{i=1}^{l}{r}_{ik}^{2}\,k=1,2,\ldots ,l$$

where ${{r}}_{{ik}}=\sqrt{{{\lambda }}_{{i}}}{v}_{{ik}}$, *λ*_*i*_ is the *ith* eigenvalues of Σ and ${v}_{ik}$ is the *kth* value of *ith* eigenvector of Σ.

### Fast density peak-based clustering (FDPC)

FDPC^36^ utilizes the two reasonable assumptions to create a metric to describe the importances of different spectrum bands. A good examplar should has high local density and relatively large distance from points of higher density^56^. It regards all the pixel values of the *ith* band, as a new data, noted as *s*_*:i*_. The local density of the *ith* band, and its relatively distance to the higher density are described by *ld*_*i*_ and *dh*_*i*_, as shown in Equation 6.

6 $$\{\begin{array}{c}l{d}_{i}=\sum _{j=1}^{l}\chi ({d}_{ij}-{d}_{c})\\ d{h}_{i}=\mathop{{\rm{\min }}}\limits_{j:l{d}_{j} > l{d}_{i}}({d}_{ij})\end{array}$$

where *d*_*ij*_ is the Euclidean distance (2-norm operator) between the *ith* specturm band *s*_*:i*_ and *jth* specturm band *s*_*:j*_, defined as ${d}_{ij}={\Vert {s}_{:i}-{s}_{:j}\Vert }_{{2}}$. *χ* is a function of the difference value between *d*_*ij*_ and the cutoff distance *d*_*c*_. If its input is negative, the function value is 1. Otherwise, the function value is 0. In FDPC, the importances of each band are defined as Equation 7.

7 $$Weigh{t}_{k}={\sum }_{k=1}^{l}l{d}_{k}\,\ast \,d{h}_{k}\,k=1,2,\ldots ,l$$

### Similarity-based unsupervised band selection (SUBS)

SUBS^35^ is a sequential forward search algorithm to achieve band selection. The algorithm starts from two initial bands determined by the maximum projection algorithm. Then SUBS assumes all other bands can be estimated linearly by the existing bands. The new selected band should lead to the largest prediction error. The process continous until the number of selected bands meet the target.

## Data Availability

The data that support the findings of this study are available from the corresponding author upon reasonable request.
